# Interacting and joint effects of triglyceride-glucose index and body mass index on future endometrial cancer risk and the mediating role of body mass index: a cross-sectional study

**DOI:** 10.3389/fendo.2025.1571693

**Published:** 2025-06-05

**Authors:** Yixiao Wang, Wenzhi Kong, Yuning Geng, Chunyu Xu, Jinwei Miao

**Affiliations:** ^1^ Department of Gynecologic Oncology, Beijing Obstetrics and Gynecology Hospital, Capital Medical University, Beijing Maternal and Child Health Care Hospital, Beijing, China; ^2^ Laboratory for Clinical Medicine, Capital Medical University, Beijing, China

**Keywords:** endometrial cancer, metabolic syndrome, triglyceride-glucose index, insulin resistance, body mass index, mediation effect

## Abstract

**Background:**

The triglyceride-glucose (TyG) index, a promising biomarker for insulin resistance, is linked to the risk of various metabolic-related cancers. However, to date, data on the association of TyG index with different subtypes of endometrial cancers and the potential mediating role of clinical factors remain limited.

**Methodology:**

Data was collected from the Beijing Obstetrics and Gynecology Hospital, Capital Medical University spanning from 2014 to 2024. Female participants with complete data on the TyG index were included in the analysis. Multivariate logistic regression analyses were used to assess the association of TyG index with endometrial cancer risk, adjusted by series of confounders. Mediation effects were evaluated using Valeri and VanderWeele’s method.

**Results:**

A total of 1,194 eligible participants were enrolled, with 597 (50%) women diagnosed with endometrial cancer. The fully adjusted multivariate logistic regression model revealed a significant association between the TyG index and the risk of endometrial cancer (odds ratio [OR]_Tertile 3 versus Tertile 1_: 1.93, 95% confidence interval [CI]: 1.37–2.73; *P* for trend=0.028). Notably, BMI exhibited significant mediation effects on this association, even after adjusted by potential confounders (*P* for trend<0.001). The proportion of the effect mediated by BMI was 25% in the crude analysis (95% CI: 0.15, 0.37; *P*<0.001) and increased to 41% in the adjusted analysis (95% CI: 0.24, 0.76; *P*<0.001). However, no correlation was found between TyG index and the clinical characteristics of endometrial cancer (*P*>0.05). Moreover, BMI is associated with the risk of different endometrial cancers.

**Conclusion:**

This cross-sectional study demonstrated that a higher TyG index, representing higher level of insulin resistance, was associated with a higher risk of endometrial cancer. Importantly, we found that BMI acted as a significant mediator in this relationship. Prospective studies are needed to further validate these findings.

## 1 Introduction

Endometrial cancer (EC), as the fourth most common female malignancy in high socioeconomic index nations and the sixth most common cancer in women worldwide, accounted for about 417,000 newly diagnosed cases globally in 2022 (1). Early diagnosis is associated with favorable prognostic outcomes. Various clinicopathological variables were identified as the risk factors for EC, including advanced age, tumor grade, size, histological subtype, the depth of uterine myometrial invasion, the cervical stromal involvement, estrogen receptor (ER) and progesterone receptor (PR) expression, and lymphovascular space invasion (LVSI) (2). These factors can be stratified into categories of low, low-intermediate, high-intermediate, or high risk for recurrence (3). Besides, with the development of deep research to genetic level, molecular classification offers an objective and reproducible framework for categorizing ECs, thereby facilitating prognosis and guiding therapeutic decision-making (4).

The coexistence of obesity, diabetes, and hypertension, commonly termed the “triple syndrome” of EC, is frequently observed in patients with metabolic syndrome. Insulin resistance serves as a pivotal component of metabolic syndrome and a common pathophysiological characteristic of the triple syndrome, heightens the likelihood of developing EC (5). The Triglyceride-Glucose index (TyG index), is considered a surrogate marker for insulin resistance (6). Due to its accessibility and cost-effectiveness, the association between TyG index and various diseases has been increasingly explored, including cardiovascular disease (7), non-alcoholic fatty liver disease (8) and Post-COVID-19 syndrome (9). A study analyzing the NHANES database suggested that the TyG index may emerge as a promising biomarker for the female reproductive cancers in American population (10). However, a retrospective study indicated that TyG index may serve as a potential marker for EC progression, with higher TyG index correlating with more advanced stages (11). However, this remains in the early stages of investigation, with several research gaps yet to be addressed, including the potential association of the TyG index with clinicopathological characteristics of EC.

Therefore, we conducted this study to investigate the predictive value of the TyG index on the risk of EC and its association with EC characteristics, based on a Chinese cohort. Meanwhile, we sought to explore the factors that mediate the relationship between the TyG index and EC.

## 2 Materials and methods

### 2.1 Study population

In this cross-sectional study, we recruited patients who diagnosed with EC or benign diseases (such as endometrial polyps and endometrial inflammation) at Beijing Obstetrics and Gynecology Hospital of Capital Medical University, spanning from 2014 to 2024. Our inclusion criteria ensured that patients had comprehensive data. This study received formal approval from the Ethics Committee of Beijing Obstetrics and Gynecology Hospital of Capital Medical University (approval number: 2024-KY-111-01). And this study utilized anonymized clinical data collected during routine patient care. Meanwhile, no additional interventions or sampling were performed for research purposes. Therefore, an exemption was granted for the requirement of informed consent, as deemed appropriate by the ethics committee.

Based on pathology results, we categorized all participants into two groups: the EC group and the non-EC group. Furthermore, in the EC patient cohort, we subdivided them into two histological subgroups: endometrioid cancer and non-endometrioid cancer. Adhering to the International Federation of Gynecology and Obstetrics (FIGO) 2009 guidelines, EC patients were stratified into early-stage (FIGO I) and advanced-stage (FIGO II or higher). Additionally, we classified EC patients into four subtypes, POLE ultramutated (POLEmut), Mismatch Repair deficiency (MMRd), p53 abnormality (p53abn), No Specific Molecular Profile, (NSMP), according to the WHO molecular classification and previous study (12). Meanwhile, the data on LVSI, depth of myometrial invasion, lymph node metastasis status, ER and PR expression were included in the EC group. The histological subtypes of EC, the presence of LVSI, lymph node metastasis, myometrial invasion, and the expression of ER and PR were all determined based on pathological assessments.

### 2.2 Covariates

Based on the previous research, we identified the following covariates of both EC and non-EC groups in our analysis: age (years), body mass index (BMI), menopausal status (yes or no), hypertension (yes or no), diabetes (yes or no), gravidity (number of pregnancies), and parity (number of live births). We gathered data on age, gravida and parity history, weight, height, as well as fasting blood glucose and triglyceride levels from clinical records, prior to any surgical interventions or pharmacological treatments. BMI was computed directly using the formula (BMI = weight (kg) / height (m^2^)), where weight and height measurements were taken by medical professionals. Furthermore, the TyG index was calculated according to a previously established method (TyG index = ln [fasting triglycerides (mg/dl) × fasting glucose (mg/dl) / 2]) (6).

### 2.3 Statistical analysis

Categorical variables were analyzed using the χ² test, while continuous variables were evaluated through t-test or the Wilcoxon rank sum nonparametric test, based on the results of normality assessment. To assess the relationship between the TyG index and EC, we employed multivariate logistic regression models to calculate ORs and 95% CIs. In Model 1, we solely considered the association between TyG index and the EC risk without adjusting for any covariates. Model 2 was adjusted for age and BMI. Subsequently, we incorporated menopause status, hypertension, diabetes, gravidity, and parity into Model 3. Additionally, to explore the potential mediator on the association between TyG index and EC, we conducted mediation analyses. The mediation effect was evaluated using the method proposed by Valeri and VanderWeele (27), which allowed for the decomposition of the total effect into direct and indirect effects, and accounted for exposure-mediator interactions. The CI was estimated using a bootstrapping approach with 1000 repetitions. Furthermore, we also applied binary logistic regression to investigate indicators related to the pathological characteristics of EC.

All statistical analyses were performed using R software (version 4.2.2) and SPSS (version 27). Statistical significance was set at a two-sided *P* value of less than 0.05.

## 3 Results

### 3.1 Characteristics of the participants

**Table 1** presents the baseline characteristics of the EC and non-EC groups. Among the 1,194 eligible participants, including 597 EC participants and 597 non-EC participants. Among the 597 EC patients, including 519 endometrioid cancer and 78 non-endometrioid cancer, 443 were early-stage (FIGO I), while 154 were categorized as advanced-stage (FIGO II or higher). The EC cohort included POLEmut (n=40), MMRd (n=115), p53abn (n=64), and NSMP (n=378). A total of 188 patients with positive LVSI and 409 with negative LVSI were enrolled in the EC cohort. Additionally, 66 EC patients were found to have lymph node metastasis, while 554 patients were tested positive for ER and 541 were positive for PR.

**Table 1:** Baseline characteristics between endometrial cancer and non-endometrial cancer.

Characteristics	Overall (n=1194)	Non-endometrial cancer (n=597)	Endometrial cancer (n=597)	*P*
Age, mean (SD)	50.00 (10.16)	45.76 (9.16)	54.23 (9.32)	<0.001
BMI, mean (SD)	25.51 (4.40)	24.40 (3.92)	26.61 (4.58)	<0.001
Hypertension, n (%)				<0.001
No	828 (69.3)	494 (82.7)	334 (55.9)	
Yes	366 (30.7)	103 (17.3)	263 (44.1)	
Diabetes, n (%)				<0.001
No	1039 (87.0)	560 (93.8)	479 (80.2)	
Yes	155 (13.0)	37 (6.2)	118 (19.8)	
Menopausal status, n (%)				<0.001
No	710 (59.5)	461 (77.2)	249 (41.7)	
Yes	484 (40.5)	136 (22.8)	348 (58.3)	
Pregnancy (number), n (%)				<0.001
0	120 (10.1)	65 (10.9)	55 (9.2)	
1	274 (22.9)	179 (30.0)	95 (15.9)	
2	330 (27.6)	156 (26.1)	174 (29.1)	
≧3	470 (39.4)	197 (33.0)	273 (45.7)	
Parity (number), n (%)				<0.001
No	173 (14.5)	97 (16.2)	76 (12.7)	
1	705 (59.0)	408 (68.3)	297 (49.7)	
2	254 (21.3)	85 (14.2)	169 (28.3)	
≧3	62 (5.1)	7 (1.2)	55 (9.3)	
TyG index, (mean (SD)	8.67 (0.59)	8.51 (0.56)	8.82 (0.59)	<0.001
Tertiles of TyG index				<0.001
Tertile 1	398 (33.3)	270 (45.2)	128 (21.4)	
Tertile 2	398 (33.3)	193 (32.3)	205 (34.3)	
Tertile 3	398 (33.3)	134 (22.4)	264 (44.2)	

BMI, Body Mass Index; TyG, Triglyceride-Glucose; SD, Standard Deviation.

The average age of the total study population was 50 years, ranging from 22 to 83 years. The mean BMI was 25.51 kg/m², and those in the EC group had a higher BMI (26.61 kg/m² vs. 24.40 kg/m², *P*<0.001). EC patients were more likely to have hypertension (44.1% vs. 17.3%, *P*<0.001) and diabetes (19.8% vs. 6.2%, *P*<0.001) compared to the non-EC group. Additionally, EC patients were more likely to be menopausal.

The mean TyG index for all participants was 8.67. Notably, the mean TyG index was significantly higher in the EC group compared to the non-EC group (8.82 vs. 8.51, *P*<0.001). Furthermore, a higher proportion of participants in the highest tertile (Tertile 3) of the TyG index was observed in the EC group (44.2% vs. 22.4%), while a lower proportion was found in the lowest tertile (Tertile 1) (21.4% vs. 45.2%).

### 3.2 The association of TyG index and EC

The relationships between the TyG index and EC risk in the entire cohort are detailed in **Table 2**. In the unadjusted model, participants in the highest tertile of the TyG index had an odds ratio of 4.28 (95% CI: 3.2-5.73) for EC compared to those in the lowest tertile, with a significant trend (*P*<0.001). This positive association between the TyG index and EC risk remained consistent in the adjusted multivariate models 2 and 3. After accounting for potential confounders, individuals in the highest tertile of the TyG index had a 93% increased risk of EC compared to those in the lowest tertile (OR _Tertile 3 vs. Tertile 1_: 1.93, 95% CI: 1.37-2.73). Furthermore, across all models, there was a significant upward trend in the adjusted ORs of EC with increasing tertiles of the TyG index (Model 1: *P*<0.001, Model 2: *P*<0.001, Model 3: *P*=0.028).

**Table 2:** Associations between the TyG index and endometrial cancer in the total cohort.

TyG index	OR(95%CI)		
	Model 1^a^	Model 2^b^	Model 3^c^
Continuous values	2.6 (2.09-3.23)*	1.46 (1.14-1.86)*	1.42 (1.11-1.82)*
(6.586-12.220)			
Tertile categories			
T1 (6.586-8.396)	Reference	Reference	Reference
T2 (8.396-8.889)	2.24 (1.68-2.97)	1.50 (1.08-2.06)	1.44 (1.04-1.99)
T3 (8.889-12.220)	4.28 (3.2-5.73)*	2.03 (1.45-2.85)*	1.93 (1.37-2.73)*
*P* for trend	<0.001	<0.001	0.028

*Statistically significant association.

a Model 1 was adjusted for nothing.

b Model 2 was adjusted for age, body mass index.

c Model 3 included the covariates of Model 2 with additional adjustment for menopausal status, hypertension, gravidity, and parity.

TyG index, Triglyceride-glucose index; OR, Odds Ratio; CI, Confidence Interval.

When evaluating the association between the TyG index and EC risk on a continuous scale, the fully adjusted logistic regression model showed a positive relationship between the TyG index and EC (OR: 1.42, 95% CI: 1.11-1.82).

### 3.3 The mediation effect of BMI between TyG index and EC

**Figure 1:**
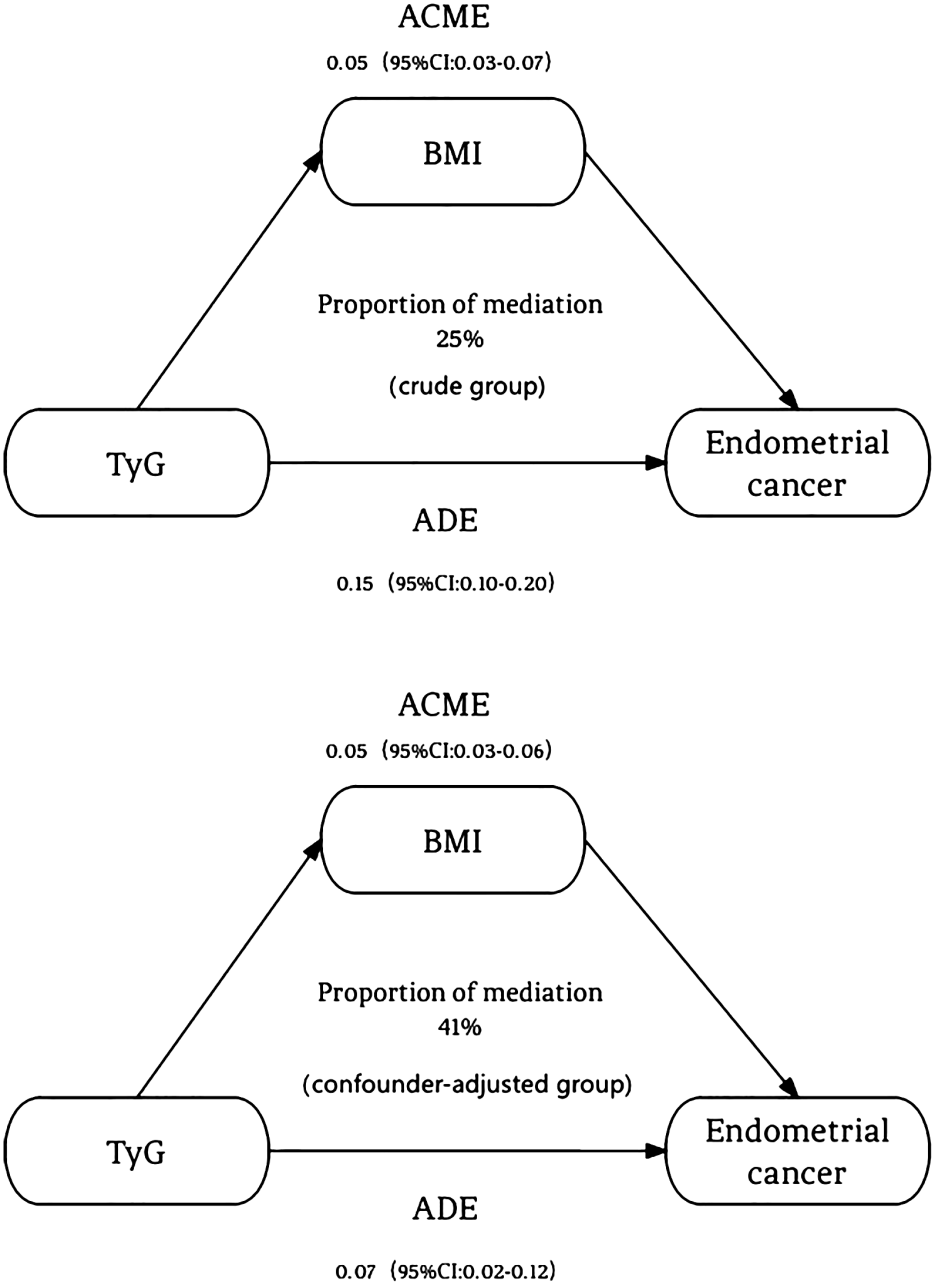
The mediation effect of BMI between TyG index and endometrial cancer. The figure revealed significant Average Causal Mediation Effects (ACME), Average Direct Effects (ADE), and total effects. Even after adjusting for potential confounders, these effects remained statistically significant (*P*<0.001). Specifically, the proportion of the effect mediated was 25% in the crude group (95% CI: 0.15, 0.37; *P*<0.001) and increased to 41% in the confounder-adjusted group (95% CI: 0.24, 0.76; *P*<0.001). ACME, Average Causal Mediating Effect; CI, Confidence Interval; BMI, Body Mass Index; TyG, Triglyceride-Glucose; ADE, Average Direct Effect.

**Figure 2:**
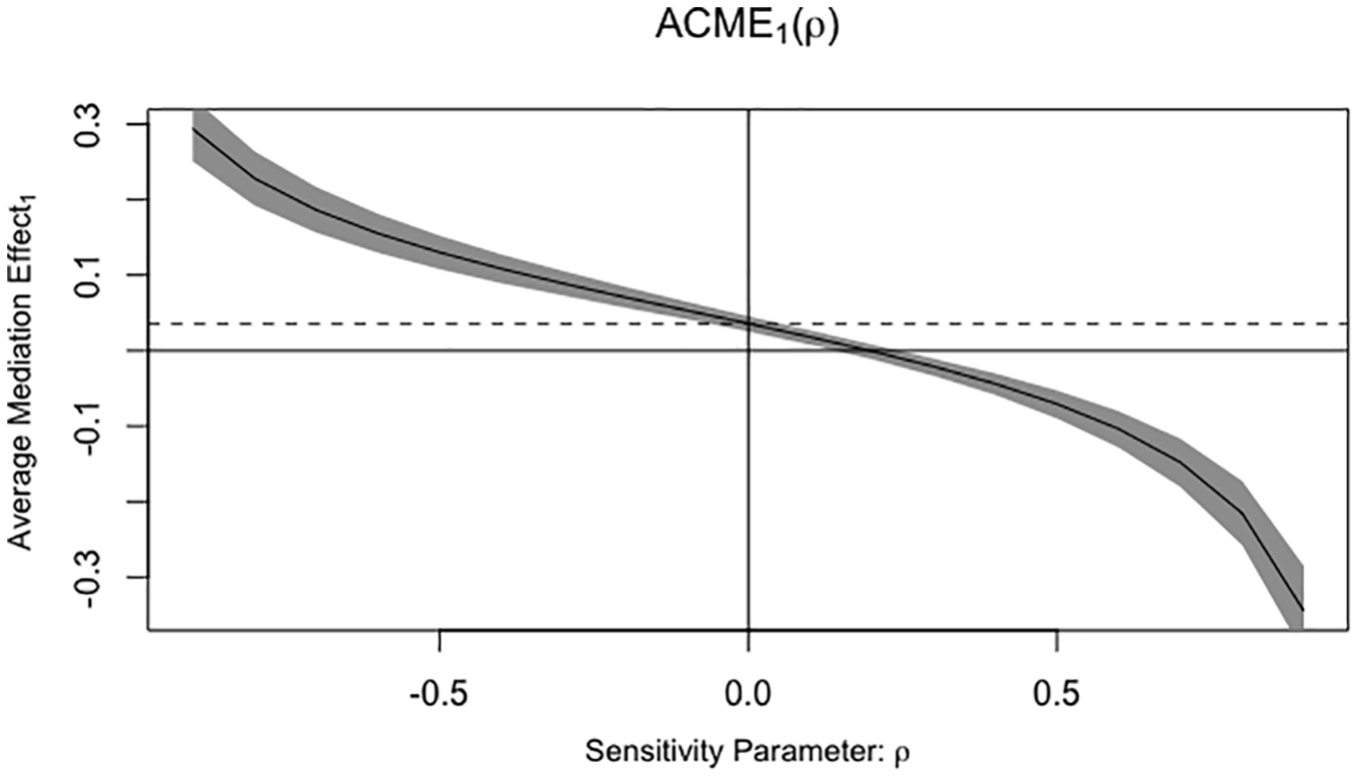
Sensitivity analysis and the rho values. ACME, Average Causal Mediating Effect; ρ, Residual correlation coefficient.

The **Figure 1** illustrates our causal mediation analysis, which employed nonparametric bootstrap CIs using the percentile method. Our findings revealed significant Average Causal Mediation Effects (ACME), Average Direct Effects (ADE), and total effects. Even after adjusting for potential confounders, these effects remained statistically significant (*P*<0.001). Specifically, the proportion of the effect mediated was 25% in the crude group (95% CI: 0.15, 0.37; *P*<0.001) and increased to 41% in the confounder-adjusted group (95% CI: 0.24, 0.76; *P*<0.001). Notably, the mediation effect was higher in the EC group compared to the non-EC group (0.28 vs. 0.22 and 0.42 vs. 0.39, respectively). This analysis was conducted using a sample size of 1194, with 1000 simulations performed to ensure robustness. Additionally, we conducted a sensitivity analysis, which also yielded significant results, and the rho values are presented in the **Figure 2**.

### 3.4 The association of TyG index and EC characteristics

To investigate the relationship between the TyG index and the characteristics of EC (such as FIGO stages, molecular classifications, presence of myometrial invasion, etc.), we conducted comprehensive subgroup analyses. However, despite adjusting for potential confounders, we did not observe any significant associations (*P*>0.05). Results are shown in the **Table 3**.

**Table 3:** Correlation analysis between TyG index and pathological features of endometrial cancer.

Pathological feature	Regression coefficient	OR	95% CI	P
Histological type				0.055
Endometrioid cancer(519)	Reference	Reference	Reference	
Non-endometrioid cancer(78)	-0.42	0.657	0.428-1.009	
FIGO stage				0.959
FIGO I(443)	Reference	Reference	Reference	
FIGO II or higher(154).	-0.008	0.992	0.727-1.354	
Myometrial invasion				
<1/2	Reference	Reference	Reference	0.172
≧1/2	0.2	1.221	0.917-1.626	
LVSI				0.886
without	Reference	Reference	Reference	
with	-0.021	0.979	0.730-1.312	
lymphatic metastasis				0.530
without	Reference	Reference	Reference	
with	0.139	1.149	0.745-1.771	
Molecular classifications				
MMRd(n=115)	-0.081	0.922	0.498-1.705	0.795
p53 abn(n=64)	-0.28	0.756	0.385-1.484	0.416
NSMP(n=378)	-0.096	0.908	0.520-1.587	0.735
POLE mut(n=40)	Reference	Reference	Reference	Reference

TyG, Triglyceride-Glucose; OR, Odds Ratio; CI, Confidence Interval; FIGO, International Federation of Gynecology and Obstetrics; LVSI, Lymphovascular Space Invasion; ER, Esterogen Receptor; PR, Progesterone Receptor; NSMP, No Specific Molecular Profile; MMRd, Mismatch Repair Deficiency.

**Figure 3:**
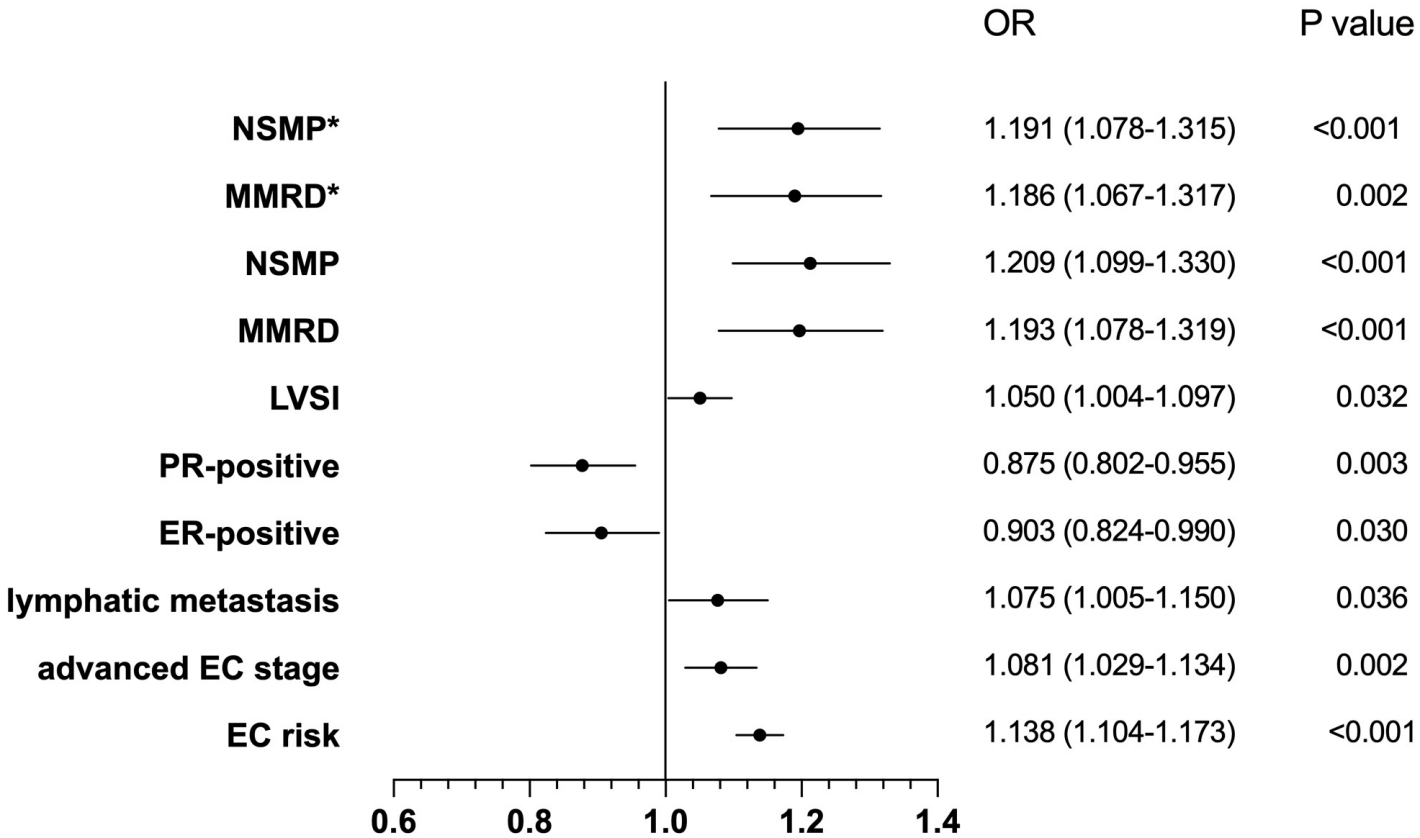
The correlation between BMI and the characteristics of endometrial cancer. OR, Odds Ratio; CI, Confidence Interval; NSMP, No Specific Molecular Profile; MMRD, Mismatch Repair Deficiency, MMRd; LVSI, Lymphovascular Space Invasion; PR, Progesterone Receptor; ER, Esterogen Receptor; EC risk, Endometrial cancer risk. *represents adjusting for potential confounders.

On the other hand, our fully adjusted logistic regression models unveiled a statistically notable positive correlation between BMI and the risk of EC with an OR of 1.138 (95% CI: 1.104-1.173; *P*<0.001), and the correlation also existed within specific subgroups. More precisely, our findings indicated that BMI was linked to an elevated risk among participants diagnosed with advanced-stage EC, with an OR of 1.081 (95% CI: 1.029-1.134; *P*=0.002). This positive association was also observed in individuals with lymph node metastasis, yielding an OR of 1.075 (95% CI: 1.005-1.150; *P*=0.036). Furthermore, BMI was inversely associated with the risk of ER-positive EC patients (OR 0.903, 95% CI: 0.824-0.990; *P*=0.030), and similarly in those with PR-positive status (OR 0.875, 95% CI: 0.802-0.955; *P*=0.003). Notably, BMI was also associated with a heightened risk in EC patients with LVSI(OR 1.050, 95% CI: 1.004-1.097; *P*=0.032). Regarding the WHO molecular classification, BMI was significantly associated with the risk of MMRd subtype (OR 1.193, 95% CI: 1.078-1.319; *P*<0.001) and NSMP subtype (OR 1.209, 95% CI: 1.099-1.330; *P*<0.001), compared to POLEmut subtype. After adjusting for potential confounders, these associations remained significant (MMRd: OR 1.186, 95% CI: 1.067-1.317; *P*=0.002; NSMP: OR 1.191, 95% CI: 1.078-1.315; *P*<0.001), as shown in the **Figure 3**.

Furthermore, we explored the relationship between WHO molecular classification and pathological features of EC, results are shown in **Table 4**. The results indicated that P53abn EC were more frequently linked to lymph node metastasis, with an OR of 4.826 (95% CI: 1.320-17.649; *P*=0.017). Additionally, P53abn EC were inversely associated with ER-positive status (OR 0.043, 95% CI: 0.006-0.331; *P*=0.003) and PR-positive status (OR 0.162, 95% CI: 0.052-0.511; *P*=0.002). On the other hand, the NSMP subtype showed a significant association with PR-positive status (OR 3.389, 95% CI: 1.039-11.053; *P*=0.043) and myometrial invasion (OR 3.311, 95% CI: 1.449-7.564; *P*=0.005).

**Table 4:** Correlation analysis between molecular classification and pathological features of endometrial cancer.

Molecular classifications	Pathological feature	*P*	OR	95% CI
p53 abn	Lymph nodemetastasis	0.017	4.826	1.320-17.649
	ER positive	0.003	0.043	0.006-0.331
	PR positive	0.002	0.162	0.052-0.511
NSMP	PR positive	0.043	3.389	1.039-11.053
	Myometrial invasion	0.005	3.311	1.449-7.564

OR, Odds Ratio; CI, Confidence Interval; ER, Esterogen Receptor; PR, Progesterone Receptor; NSMP, No Specific Molecular Profile.

## 4 Discussion

This cross-sectional study identified a significantly positive association of the TyG index with the risk of EC in a Chinese cohort, highlighting the clinical role of the TyG index on the prevention and management strategies for EC. BMI mediated this association, indicating that the TyG index increases EC risk by affecting BMI, which may explain the previous research that why the mediation effect of TyG between BMI and cancer risk was not observed for EC (13).

Metabolic syndrome is consistently associated with an increased risk and the poorer prognosis of EC (14, 15). Insulin resistance, a pivotal underlying mechanism in metabolic syndrome, could directly and indirectly impact EC development. The direct mechanism involves the activation of key signaling pathways, including PI3K/Akt and Ras/MAPK, as well as signaling pathway crosstalk among insulin, IGF-1, and estrogen. In the indirect mechanism, excess insulin could result in low levels of blood sex hormone-binding globulin as well as high levels of blood estrogen and androgen, which promote EC development (16–19). The TyG index has been recognized as a promising surrogate measure of insulin resistance and reported to be associated with the risk of various metabolic-related diseases, including EC. In our study, we also found a positive association of TyG index with EC risk. Meanwhile, we investigated the association between the TyG index and clinicopathological characteristics of EC, such as histological type, FIGO stages, LVSI, molecular classification. However, no significant results were found, indicating that the TyG index is a good predictor of EC risk but cannot distinguish different EC features. These findings further indicate that insulin resistance may represent a shared pathological feature across different histological subtypes of EC. The growing prevalence of obesity is the primary cause of EC, with a 54% higher risk of EC cancer per 5 kg/m² increase in BMI (20, 21). The underlying role of obesity in the pathogenesis of EC remains incompletely elucidated. Some research conveyed that obesity could lead to a proinflammatory environment dominated by high levels of C-reactive protein, interleukin-6, and tumor necrosis factor-α, as well as a relative deficiency of protective immune cell types in the endometrium, which may contribute to EC risk (22, 23). In our study, we used BMI as a surrogate marker for obesity and found that EC patients with higher BMI were more likely to have lymph node metastasis, negative ER and PR expression, and deeper LVSI, which commonly predicts poor prognosis of EC.

Furthermore, at the genetic level, different molecular subtypes indicates different prognosis (24). Patients with POLEmut EC tend to be relatively young with a normal BMI. POLEmut ECs are associated with a favorable prognosis despite often having high-risk pathological features, such as a high tumor grade and extensive LVSI (25, 26). MMRd ECs are often related to high-grade EC. NSMP groups encompass mostly endometrioid carcinomas with high expression of ER and PR and associated with an increased BMI (27). EC patients with p53abn subtype are older, and are not associated with an increased BMI. In our study, compared to POLEmut EC, patients with higher BMI are more likely to be classified as MMRd and NSMP subtypes, with intermediate prognosis and potential eligibility for immunotherapy (28). Moreover, EC patients with different molecular classifications exhibit distinct pathological features (29). In this research, patients with p53abn are more likely to have lymph node metastasis, ER negativity, and PR negativity. Meanwhile, the patients with NSMP type tend to be PR positive but exhibit deeper myometrial invasion.

Several limitations of the present study should be considered. First, the TyG index was measured through a single fasting blood sample, which may not capture longitudinal metabolic variations or account for intra-individual fluctuations in insulin resistance biomarkers. Second, Although the association between TyG index and EC risk was adjusted for various crucial confounders (age, BMI, menopausal status, hypertension, diabetes, gravidity, and parity). However, the lifestyle factors such as diet, physical activity, and genetic predispositions were not included in this study. Future studies should incorporate comprehensive lifestyle and genetic data to better control for these confounders. Third, while BMI partially mediated the TyG index-EC association, the precise biological pathways (e.g., chronic inflammation, sex hormone alterations) remain uncharacterized. Advanced techniques such as multi-omics profiling or mendelian randomization could elucidate these mechanisms. Fourth, these findings from a single-center and retrospective study may has constraints in terms of generalizability due to potential biases in patient selection, local practices, and limited demographic diversity, multi-center and prospective studies are needed in the future.

## 5 Conclusion

This cross-sectional study demonstrated that a higher TyG index, representing higher level of insulin resistance, was associated with a higher risk of EC. Importantly, we found that BMI acted as a significant mediator in this relationship. Prospective studies are needed to further validate these findings.

## Data Availability

The original contributions presented in the study are included in the article/supplementary material. Further inquiries can be directed to the corresponding author.
